# 165. Emergence of Extensively Drug-Resistant *Salmonella enterica* Serotype Typhi Infections—United States, 2008–2020

**DOI:** 10.1093/ofid/ofab466.165

**Published:** 2021-12-04

**Authors:** Felicita Medalla, Louise Francois Watkins, Michael Hughes, Meseret Birhane, Layne Dorough, Chelsey Griffin, Jared Reynolds, Hayat Caidi, Hattie E Webb, Eric Mintz, Bruce Gutelius, Gayle Langley

**Affiliations:** 1 Centers for Disease Control and Prevention, Atlanta, GA; 2 Division of Foodborne, Waterborne, and Environmental Diseases, Centers for Disease Control and Prevention, Atlanta, Georgia; 3 Division of Foodborne, Waterborne, and Environmental Diseases, Centers for Disease Control and Prevention, Atlanta, GA, Atlanta, Georgia

## Abstract

**Background:**

Typhoid fever, caused by *Salmonella* Typhi, is fatal in 12%–30% of patients not treated with appropriate antibiotics. In 2016, a large outbreak of extensively drug-resistant (XDR) Typhi infections began in Pakistan with cases reported globally, including the United States. In 2021, the Centers for Disease Control and Prevention (CDC) issued a health advisory on XDR infections among U.S. residents without international travel. We describe resistance of Typhi infections diagnosed in the United States to help guide treatment decisions.

**Methods:**

Typhoid fever is a nationally notifiable disease. Health departments report cases to CDC through the National Typhoid and Paratyphoid Fever Surveillance system. Isolates are submitted to the National Antimicrobial Resistance Monitoring System for antimicrobial susceptibility testing (AST) using broth microdilution. AST results are categorized by Clinical and Laboratory Standards Institute criteria. We defined XDR as resistant to ceftriaxone, ampicillin, chloramphenicol, and co-trimoxazole, and nonsusceptible to ciprofloxacin.

**Results:**

During 2008–2019, of 4,637 Typhi isolates, 52 (1%) were ceftriaxone resistant (axo-R); 71% were ciprofloxacin nonsusceptible, 1 azithromycin resistant (azm-R), and none meropenem resistant. XDR was first detected in 2018, in 2% of 474 isolates and increased to 7% of 535 in 2019. Of the 52 axo-R isolates, 46 were XDR, of which 45 were from travelers to Pakistan, and one from a non-traveler; 6 were not XDR, of which 4 were linked to travel to Iraq. In preliminary 2020 reports, 23 isolates were XDR; 14 were from travelers to Pakistan, 8 from non-travelers, and 1 from someone with unknown travel status. Among those with XDR infection, median age was 11 years (range 1–62), 54% were female, and 62% were from 6 states.

**Conclusion:**

Ceftriaxone-resistant Typhi infections, mostly XDR, are increasing. Clinicians should ask patients with suspected Typhi infections about travel and adjust treatment based on susceptibility results. Carbapenem, azithromycin, or both may be considered for empiric therapy of typhoid fever among travelers to Pakistan or Iraq and in uncommon instances when persons report no international travel. Ceftriaxone is an empiric therapy option for travelers to countries other than Pakistan and Iraq.

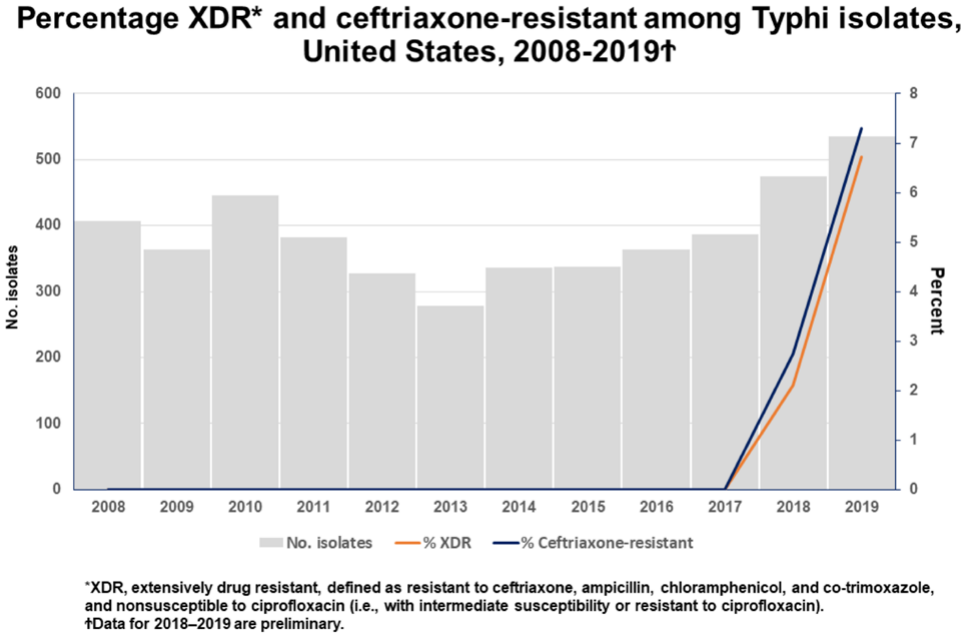

**Disclosures:**

**All Authors**: No reported disclosures

